# The Nursing Supplement

**Published:** 1890-02-01

**Authors:** 


Being the Extra Nursing Supplement of "The Hospital" Newspaper.

All Contributions for this Supplement should be addressed to the Editor, The Hospital, 139-140, Salisbury Court, Fleet Street, London, E.C., and should have the word "Nursing" plainly written in left-hand top corner of the envelope.

### En Passant.

Ashton Nursing Association.—Mrs. Buckley presided at the annual meeting of this association on January 13. During 1888 there were 162 patients on the books, who were attended by the two nurses—Nurse Howlett, who trained at Guy's, and Nurse Maud Hurcum, who trained at Bristol Royal Infirmary. There is plenty of work for a third nurse at Ashton-under-Lyne if only the funds were forthcoming.

Frome Nurses' Home.—The annual report of this charity tells of 192 patients attended during the year, 63 labourers, 58 mechanics, 44 paying patients, nine were nursed in a country village, and seven paid a small sum weekly for attendance. Good certificates as to the nurses have been received from employers; and the district nursing of the sick poor in the town and neighbourhood, together with the work of the Mothers' Aid Branch, has been carried on satisfactorily. The fees received for nursing amount to £283 12s. 3d., showing an increase of £83 12s. 3d. There has been a larger demand for nurses than could on every occasion be supplied; and, on this account, the committee desire to increase the number of their staff, for which purpose additional house-room is necessary. There are at present seven nurses, and Miss Briggs says there is not room for more without an enlargement of the home. The committee propose to go forward without hesitation.

"The Nightingale."—This American paper for nurses reappears once more under the editorship of Sarah E. Post, M.D. It tells of post-graduate classes for nurses, both the Babies' Hospital and the Women's Hospital offering to certificated nurses practice in their speciality, while the Bellevue School will take back any of its graduates for a limited time if desired. This is a capital plan and worthy of imitation. Which of our London hospitals will offer like advantages to English nurses? The St. Barnabas Guild of New York is starting a clubhouse for nurses, the members themselves subscribing towards the project. Also the nurses of St. Luke's Hospital, Chicago, are collecting money to endow a small ward for private nurses when ill. The room is to be called the Blue Cross Ward. There seems to be a great deal more enterprise and good-fellowship amongst nurses in America than there is here; but perhaps now the club in Buckingham-street is to be open daily closer union will grow amongst London nurses.

More Nurses or Higher Pay.—Those who notice our advertisement columns must be struck by the number of vacancies advertised, and the few appointments wanted. There can be no doubt that a trained nurse can easily get work just now; but she cannot so easily get a fair wage. Three years' training entitles a woman to more than £20 at the end thereof, and we hope that soon it will be more usual to offer a trained nurse £30. The demand is so much greater than the supply just now that an advertisement for a nurse at wages of £20 only brings three or four answers, and these, as a rule, are not from thoroughly trained women. Far be it from us to make nurses discontented with their wages, their thoughts have little time to dwell upon such things; but let us take thought for them, that, at least, they have fair play. "Thou wilt never sell thy life, or any part of thy life in a satisfactory manner. Give it like a royal heart, let the price be nothing: thou hast then, in a certain sense, got all for it."

Mary Adelaide Letter.—Miss Twining writes the sixteenth quarterly letter to the Mary Adelaide nurses, taking for her subject the rise and progress of the Workhouse Infirmary Nursing Association. Amongst the appointments filled by these nurses during the last quarter are a nurse and midwife to Rugby, Basingstoke, and Ormskirk. Altogether 13 nurses and five midwives were supplied by the society during three months.

Central Sick Asylum.—The annual entertainment of the Central London Sick Asylum was held on January 9th, and an excellent concert was given under the management of the chaplain, Mr. Baxter. Amongst the performers were Mr. Herbert Thorndike and Miss Maude Chichester. The patients enjoyed the entertainment very much, and so did Nurse Taylor and Nurse Chapman, who have had the nursing of the asylum ever since its commencement.

Sheffield Union.—We are very glad to b able to state that the infirmary connected with Sheffield Union is to have a thoroughly efficient nursing staff to take charge of the sick within its walls. The new scheme will come into operation on March 1st, when ten charge nurses (two for night duty), all certificated, six assistant nurses, and nine probationers will commence work in the wards. The time tables for these nurses have been carefully drawn up, and they will all have ample time off duty. Uniform will be worn, and lectures and classes will be given to the probationers. Paupers will no longer be allowed to take any part in the nursing. There is every reason to believe that in future the nursing in Sheffield Union will equal that in any other union in the kingdom, and we heartily congratulate the committee on their excellent scheme.

Thousands of Pounds for the National Pension Fund.—We have the pleasure to acknowledge the following sums towards the £14,000 now being raised to at once bring the Donation Bonus Fund up to £40,000. We hope to be able to acknowledge contributions amounting to £1,000 every week until the £40,000 are completed. Contributions of any amount may be sent to the Editor of The Hospital, or to the Manager, National Pension Fund for Nurses, 8, King-street, Cheapside, London, E.C.:

The Second Thousand.

| | £ | s. | d. |
|---|---|---|---|
| Mr. B. Akroyd | £2 | 2 | 0 |
| Messrs. A. Anderson and Co. | 100 | 0 | 0 |
| A. H. | 100 | 0 | 0 |
| Messrs. Anderson and Wyatt | 2 | 2 | 0 |
| Messrs. A. H. Baker and Co. | 105 | 0 | 0 |
| Mr. E. Baker | 5 | 0 | 0 |
| Messrs. Baker and Sturdy | 52 | 10 | 0 |
| Mr. H. Barry, jun. | 2 | 2 | 0 |
| Messrs. R. S. Barthrop, B. Baumann, and R. Buyden (one guinea each) | 3 | 3 | 0 |
| Messrs. Bristowe Bros. | 100 | 0 | 0 |
| A Broker | 100 | 0 | 0 |
| Mr. W. P. Brown | 2 | 2 | 0 |
| Mr. H. Doughty Brown | 5 | 5 | 0 |
| Messrs. Brinsted, Bourke and Co. | 100 | 0 | 0 |
| Mr. E. Buchan | 10 | 10 | 0 |
| Messrs. Campion, Pawle, and Co. | 26 | 5 | 0 |
| Messrs. Jas. Capel and Co. | 100 | 0 | 0 |
| Messrs. S. Cawston and Co. | 10 | 10 | 0 |
| Messrs. Cazenove and Akroyds | 100 | 0 | 0 |
| Chadesworth Bros. | 21 | 0 | 0 |
| Egerton H. Clarke | 50 | 0 | 0 |
| C. M. C. | 2 | 2 | 0 |

### The Nurses' Bookshelf.

### OBSTETRIC NURSING.*

*(Continued from page lxxi.)*

The later chapters of this book contain an account of the changes undergone by various organs during pregnancy, the formation of the placenta, its weight, size, and shape, &c. This description concludes with the following excellent advice: "No opportunity of examining the normal placenta should be lost, until a thorough knowledge is acquired of its details, for after every labour it must be thoroughly examined to ascertain that complete expulsion has taken place, the retention *in utero* of small detached pieces being a fertile source of severe after-pains, fœtid discharge, and puerperal fever. The examination is most satisfactorily accomplished by floating the placenta in a basin of water." Then follow a description of the fœtal circulation. Then follow chapters on the signs and symptoms of pregnancy, and, in addition to a full description of each sign, a table of them, and the time at which each may be expected, is drawn up, and will probably prove very useful to the nurse for reference; whilst further on is another table with the same symptoms arranged under heads and sub-heads, and the nurse is told how to note these signs by inspection, palpation, and auscultation. The diseases and complications are next given, classed as those which are removed with the cause, and those that remain more or less chronic. The list of these troubles is very complete, though, of course, merely the simplest domestic remedies are suggested, and the immediate cause of each is given shortly. "A number of minor nervous conditions are frequently to be noticed, such as sleeplessness, irritability of temper, and a peculiar dry, hacking cough, which is often very troublesome. These minor nervous phenomena are much more common in the higher grades of society, and are to be treated by open-air exercise, light reading, and other forms of amusement."

When describing the child the authors give also its normal size and weight, and the nurse is told that the newly-born child will cry lustily. The bone, sutures, and fontanelles are described of the offending fœtal head, to which usually the cause of difficulty in ordinary labour may be referred; but we fear that the author's remark that "civilisation and intellectual culture have also done a good deal to increase the size of the fœtal head in both sexes," if it become generally known, will range women on the side against spread of popular education.

The advice to the nurse when summoned to an expected case is good and very minute, and contains advice like the following: "Upon being called the summons must be obeyed as quickly as possible, because, should anything untoward happen, the blame, whether deservedly or not, is sure to be laid on the expected attendant. She is clearly told with what complications she may have to cope before the arrival of the medical attendant—what she should have prepared and the arrangements she should have completed." We are surprised to see, however, even in a handbook for nurses, this statement made with regard to the use of the binder: "Its use is to give comfort to the mother and, perhaps, to a certain extent, preserve the figure, not as is erroneously, though popularly, supposed to prevent hæmorrhage." We always understood that the use of a binder, either after childbirth or after tapping for dropsy, was chiefly to prevent peritonitis, which is apt to follow the sudden and complete removal of a great weight, but that the binder, by keeping up pressure for a time, gradually accustomed the abdominal cavity to the absence of the weight. During the "puerperium" stage the two great essentials "are rest and cleanliness, in attention to which the secret of success lies. The want of care in carrying out these two principles constitutes the most fertile cause of serious complications both immediate and remote." The temperature and pulse must be looked to carefully, especially the pulse, which is a most valuable indicator. "From sixty to ninety beats per minute may be taken as the range of safety. If shortly after labour it is found to be quick—*i.e.*, over 100—hæmorrhage is to be suspected, while if the like occurs later on in the puerperium, even though unattended with an elevated temperature, inflammatory mischief is to be apprehended. As a broad rule, it may be said that if the pulse and temperature are under 100 beats and degrees respectively, the patient is in a satisfactory condition." In describing the causes of delayed labour, the authors deprecate most strongly the indiscriminate use of ergot, which the nurse should not use on her own responsibility, for ergot acts by not only causing contraction, but by changing the character of the contractions from intermittent to constant.

The remaining chapters treat of deformed pelvis and the management of such serious complications as eclampsia. Hæmorrhage may be so sudden and profuse as to be rapidly fatal to the patient, and though a nurse is not expected to treat this alarming symptom, still, in case medical help is not at hand, she should know how to act to prevent her patient from bleeding to death. "It usually comes on so suddenly, and so unexpectedly, that there is no time for consultation or deliberation, and to save the patient's life the treatment must be prompt and immediate. It is absolutely criminal for anyone to undertake the responsibility of a midwifery case who is not thoroughly competent to treat *post partum* hæmorrhage."

The care of the infant, the course to be adopted if born feeble or even apparently dead, with full directions for carrying out artificial respiration according to Schultze's, Sylvester's, or Marshall Hall's method, the feeding of the child, care of the feeding-bottles, of which three should be in use at once and which are not to be cleaned with bottle brushes, as the bristles are apt to become detached and may be sucked into the child's throat; all this and much more to the same effect may be carefully remembered with advantage. A few of the most common and domestic of infants' slight ailments are noticed, and innocent homely remedies suggested as preliminary to sending for the medical attendant if they do not succeed. The directions for giving enemata, suppositories, douches are all very clear, and will be found useful by the nurse, though of course only practice will make an adept in these matters, and the book finishes up with a very fairly complete glossary of the terms used, and index.

Altogether, we can heartily recommend this little handbook to all monthly nurses, feeling confident that even an accomplished nurse will benefit by its occasional perusal to prevent any of her knowledge becoming "rusty."

* "Handbook of Obstetric Nursing." By F. W. N. Haultain, M.D., and J. Haig Ferguson. Young J. Pentland.

### In Days of Sickness.

By a Patient.

*(Continued from page lix.)*

But all our days of sickness are not days of pain, and effort to "suffer and be strong." We have our "off" days, so to speak—days when we can chaff with the doctor and laugh with the nurse. Sick people are very like children, little things amuse them, and, alas! little things put them out. A few flowers, the kind thought of a country friend, the little delicacy brought in by the dear one whose face brightens as she learns that you have passed a better night, what power these—trifles, do you call them—have to please? No, they are not trifles, but tokens of an affection which in days of health we are too apt by our actions to hurt and to offend.

And we grow better, the fever slackens, our brain can take in outside impressions. Now comes the time to summon to our bedside those friends who, like all true friends, prove their worth in the hour of trial—books. And, just as to the sick body, a variety of food is useful—even in the first stages of the fever, beef-tea and milk, not milk alone—so for the sick mind, and it is all important to remember that the mind, to a certain extent, suffers with the body—variety of food is good. The novel, the book of travel, the magazine, the favourite poet, should all take their place by the sick bed. I am doubtful about the newspaper: it crunkles, and is unwieldy to hold; besides its contents deal too entirely with that every day out-of-door life of which at present it is a weariness to think. But cling to the friends of a lifetime, books. Perhaps eyes are weak, and hands too feeble to lift up the volume, but one is ready to employ eyes, voice, and hands in your service; and weariness, pain, discomfort may all be lulled to sleep while listening to perchance some oft-told tale, rendered musical by the tones of a dear one's voice. But a fever is not the only "ill that flesh is heir to." And beside the physician's there is the surgeon's office, and with the surgeon we become introduced to that wonderful alleviator of suffering and help to the surgeon's work—anæsthetics. But though a blessed, it is without doubt an awful thing. You are sent into an unconsciousness from which it is quite within the bounds of possibility you may not recover. But apart from this you are lying on the bed, not of sickness. Perhaps you have had an accident, broken your leg, and so shattered is the limb the surgeon decrees it must come off. An hour or so ago you were in perfect health, now you are lying face to face with death.

The doctors may make light of the operation, "'Tis their vocation, Hal"; but you, it is no light matter to you, and the prelude of the awful affair is this suffocating feeling, as the drug is held before your face and you slowly glide into unconsciousness, hearing sounds, horribly suggestive sounds; feeling touches, horribly suggestive, too, after you have lost the power of speech. Then all is a blank. But the last words you heard were the kind words of good cheer uttered by the doctor, and when, long after, you struggle back into consciousness, as the sensations of soreness and pain overpower you, the gentle hands of the nurse are busy about you, and her reassuring voice tells you that it is all over. Yes, you went to sleep (let us be euphemistic when we can) with two legs, you awake with one! How much they have done to you whilst you lay unconscious! Three or four doctors busy with hand and brain, the nurse at their beck and call, and all for the benefit of your one poor little body. That at least is the patient's view of the matter, for patients are apt to think but little of the "cause of science," without which, however, a doctor could hardly get through all his difficult and disagreeable work.

Well, the surgeon's work is practically over with his one great field day. And whilst he fares forth to "fresh fields and pastures new," you lie still, and let Nature do on you her wonderful work of healing. Ah! she heals the wounds of the body quicker than those of the mind.

But human skill has still its little part to play. There are the antiseptics, the bandages, but I must not grow too technical. Day by day the pain, that great indicator of disease, grows less. Soon comes the time when you can get up again and go about your business. The days of sickness are past. Yes, they are past, though not without leaving their "trace on heart and brain," for sickness and sorrow have opened the door of the heart to sympathy. Let in the gentle guest and give her house room, for she is an "angel unawares."

How often have you passed that great building, the hospital, as you went to your daily work? Now you give a thought—yes, many thoughts—to the sick and the suffering gathered within its wall; those walls are eloquent to hearing ears. Walk through the wards, taking with you the angel—Sympathy. For seeing eyes, what a sight is here! What a concentration of the mystery of pain and the power of sympathy! What a history of life!

And as you pass out again into the hurry and turmoil of the town, remembering that but a short time ago you, too, were lying in pain and weariness on your sick bed, will not your thankfulness take the form of a thank-offering? Will you not give of your gold for the service of man, which is the service of God?

### Examination Questions.

Very few answers were received this month, probably owing to the rush of Christmas work. The prize has been awarded to Miss Pelly, of St. Bartholomew's Hospital, whose answer will be printed next week. We have forwarded her "Ralph Ellison's Opportunity," by Leslie Keith. The question for next month is "What are the various kinds of hæmorrhage? How are they distinguished? What means are adopted to stop them?" Answers must be received at our office by February 8th.

### Everybody's Opinion.

*[Correspondence on all subjects is invited, but we cannot in any way be responsible for the opinions expressed by our correspondents. No communications can be entertained if the name and address of the correspondent is not given, or unless one side of the paper only be written on.]*

### "LADIES AND WORKHOUSE INFIRMARIES."

Miss J. Wilson, hon. sec., Workhouse Infirmary Nursing Association, 6, Adam-street, Adelphi, writes: In the report of the last meeting of the Hospitals Association there was I think a slight misunderstanding about my meaning which I am anxious to make quite clear. I am reported to have said that "ladies did not suit for nurses in workhouse infirmaries." What I said was that ladies were not suited for *small country* unions. The difference is obvious, as in the latter the duties are often too varied, the general arrangements too rough, and the position too isolated to be undertaken by any trained nurse, still less by educated gentlewomen. To take an instance, we find in some unions the nurse is not only expected to cook her own food, but her duties also include the bathing of female tramps. This misconception of the proper work of a nurse arises from a want of knowledge on the subject in the minds of many country guardians, which we trust will gradually disappear. At present, however, it is evident that small unions are not suited for ladies, though in future there is no reason why a lady should be as content in them as in cottage hospital life. Meantime, therefore, I would urge ladies to leave the more overcrowded branches of the profession and to enter our metropolitan infirmaries as sisters or nurses; in many of these the rule and system are as definite as in hospitals. There is also a sphere of work admirably suited for ladies as head nurses in the large provincial workhouse infirmaries; in these positions a trained lady's organising capacity, education, and tact are very essential, as the infirmary is seldom separate from the workhouse management, and although the number of cases is frequently from three to four hundred there is often no resident doctor. In both these spheres ladies are required, and my desire to make clear this fact must I hope serve as an excuse for the length of my letter.

### THE PENSION FUND AND ITS ENEMY.

Miss K. M. Heanley writes: As a member of the Nurses' National Pension Fund, I wish to make a few comments on an editorial appearing in a contemporary. As a regular reader of this publication since its first appearance, I have been at a loss to understand the reason of its bitter tone against a movement that has the interest of "trained nurses" wholly and solely at heart. I should be sorry to think that the editor could be influenced by the fact that the founder of this fund was deeply interested in, and gave publicity to, the fund through the pages of another paper; that, surely, is too mean and small a motive to influence the editor of a paper—said to be started for the benefit of nurses—in injuring a fund equally started for their benefit; for whose advantage but nurses could such a fund at an infinite expense of time and thought on the part of its promoter have been founded? It is paying the four merchant princes, through whose munificence it has been set on foot, a poor compliment to suppose they should not be the best and only judges through whose hands and in what way their benefits shall be conferred. There is no doubt they are well assured of the *bona fide* nature of the promoter's intentions, whose scheme they so liberally approve. We are told in this editorial "that gentlewomen of education more and more adopt this calling (of nursing), and that only such women with resources that place them far beyond all chance of want, can pay the premiums regularly." Surely the writer has failed to appreciate what "Katharine Regina" taught us so pitifully about this very class of gentlewomen, daughters of high-class professional men, formerly chiefly the governess class, often ladies whose salaries placed them in a position of independence during their working days, but left them, as Besant has shown, almost at starvation point when they could work no longer. These, the very class who now crowd into the "trained nurses" ranks, might, had the way been made clear for them, have paid such premiums to secure such pensions in their day. I would, therefore, demand that the writer of these articles no longer tries to injure a fund which, he must know, is based on purely philanthropic principles, and will be invaluable to many, even if it does not in all ways commend itself to him.

THE OLDER NURSES.

A Well-wisher writes: When the question first arose as to what are nurses of forty, fifty, and upwards to do, I waited, hoping that someone, who could express what they meant better than I, would mention that such nurses might be employed as night nurses in hospitals. They would then be sure of a comfortable home, and have light work. Five-and-twenty years ago, when I was probationer at one of the London hospitals, there were one or two nurses who would be fifty and upwards. These women were employed as night nurses. They received I believe, a salary of ten guineas a year and their uniform. Could not the so-called superannuated nurses of the present time be supplied with similar work now, especially in small hospitals where only one night nurse is employed. Would it not be much better and safer for an experienced, reliable, elderly person to hold the position of night nurse rather than a young inexperienced girl, and this apart from the temptations to which occasionally a young girl is exposed when on duty as night nurse.

### Appointments.

[It is requested that successful candidates will send a copy of their applications and testimonials, with date of election, to The Editor, The Lodge, Porchester-square, W.]

St. Pancras Infirmary.—Miss Botham has been appointed assistant matron of this infirmary.

Clifton College Sanatorium.—Miss E. S. Scoan, who trained at St. George's, has been appointed matron of this sanatorium.

Melbourne Hospital.—Miss Isabella J. Rathie, of Hobart Hospital, Tasmania, has been appointed matron of the Melbourne Hospital. There were 22 applicants for the post.

Birmingham Skin and Lock Hospital.—Miss Horner, of the East Suffolk Hospital, Ipswich, has been unanimously elected matron-nurse for the new in-patient department of the Birmingham Skin and Lock Hospital.

Bristol Royal Infirmary.—Mrs. Mathias, who has held the post of matron at the Clifton College Sanatorium for the last two years, has been appointed night superintendent at the Bristol Royal Infirmary. Mrs. Mathias was trained at St. Bartholomew's, and was afterwards sister at the Salop Infirmary. She holds excellent testimonials.

### Notes and Queries.

To Correspondents.—1. Questions may be written on post-cards. 2. Advertisements in disguise are inadmissible. 3. In answering a query please quote the number. 4. If a private answer is desired, a stamped addressed envelope must be forwarded.

Notice.—We must really ask our correspondents to be more particular in enclosing name and address. Constantly we receive queries or letters with no name attached.

### Queries.

(41) *Nursing Abroad.*—I should be glad to hear of a private nursing institution near Christchurch, New Zealand, or at Montreal. *E. S. V.*

(42) *The Royal Red Cross.*—Where can I find a complete list of those who have received this order? *Sister May.*

(43) *Medical Book.*—What is the best index book of diseases other than the Nomenclature of 1885, with aid to diagnosis? *Ataton.*

(44) *Home for Girl.*—Convalescent home wanted for girl aged 15, and subject to epileptic fits. Parents very poor. *M. A. E.*

(45) *Home for Women.*—Two sisters, aged 22 and 23, both with weak intellects and heart disease. Cannot pay, but can work. *Sister Katherine.*

(46) *Home for Incurables.*—Is there a home for incurables where a man can be received for 10s. a week? *E. C. R.*

(47) *Home for Nurse.*—Wanted, by the sea, for a tired nurse, who wants two or three weeks' rest. *Nurse Leah.*

(48) *Obstetrical Diploma.*—How can a certificated monthly nurse obtain this without going into hospital? *M. B.*

### Answers.

*Ella.*—We know nothing beyond the advertisement. The Auckland Hospital, New Zealand, is a good one; 19 nurses.

*G. C.*—Male nurses are trained by the Hamilton Association, of 22, South Audley-street, W., and Mr. Wilson's Institution, of 97, Wimpole-street, W. This query has been constantly replied to before.

*Nurse R.*—The Lying-in Hospital in question is Queen Charlotte's, but such details are also taught at Endell-street and the City-road Lying-in Hospital. Pardon delay in procuring you this information.

*Nellie.*—Parker's, of 145, Oxford-street, make a perfectly noiseless and very comfortable shoe especially for nurses. Price 9s. 6d.

*L. H. S.*—We are afraid the dolls cannot go to Gloucester

*An Old Guy's Nurse.*—We sympathise with you, but, after all, we are sure you do not envy the others their medals.

*Ataton.*—All Army and Navy nurses are ladies by birth and position, and hold a good social position. The social status of a hospital nurse varies according to her education, but it is decidedly rising. Asylum attendants take a lower social standing, chiefly because they are entirely recruited from the domestic servant class.

*H. L. A.*—We will remember your request about gardening notes, and if ever we have room will supply some

### Presentation.

Last week the nurses and servants of St. Pancras Infirmary presented Miss Parsons, the assistant matron, with a handsome service of plate. This is one of the many valuable tokens of regard Miss Parsons has received on the occasion of her marriage

### Notices.

To Our Readers.—In view of certain changes which are imminent in consequence of the large and ever-increasing circulation of The Hospital, the Editor requests every matron, sister, or nurse who takes the paper regularly to send him notice of her name and address, to The Lodge, Porchester-square, London, W. Postcards may be used.

### Vacancies.

### Matrons.

Stockport Infirmary, £60.

### Nurses.

Aylesbury Infirmary, £25.
Birmingham Orthopœdic Hospital.
Blackheath Institute.
Bournemouth Nursing Institution, £25.
Bridgewater Infirmary, £25.
Brighton Nurses' Institution.
Canterbury Union, £25.
Central London Throat Hospital, £22.
Dublin Fever Hospital, £25.
Eastbourne Home Hospital.
Frome Nurses' Home, £22.
General Nursing Institute.
Glasgow Victoria Infirmary, £24.
Hollond Institution, Nice.
Jersey Nurses' Institute, £25.
Kent Nursing Institution.
King's Cross Hospital, Dundee, £24
Leicester Institution, £25.
Leith Hospital, £25.
Levick Institution, Paris.
Montreal, Canada, £37.
North London Consumption Hospital, £24.
Nottingham Nursing Association.
Nursing Institution, 96, 97, 98, Wimpole-street, London, W., Mr. Wilson has several vacancies for thoroughly-trained nurses.
Pendlebury Hospital, £20.
Rivers-street Home, Bath.
Royal Portsmouth Hospital, £25.
Sheffield Fever Hospital, £25.
Shoreditch Infirmary, £20.
St. Peter's Institution, Bedford.
St. Saviour's Infirmary, £20.
St. Vanda's Home, Bromley, £20.
Stroud Hospital, £23.
Swansea Institute, £25.
Tunbridge Wells Hospital, £23.
Ventnor Consumption Hospital, £20.

### District Nurses.

Berkhamsted.
Frodsham, Cheshire.
Halifax.
Kensington District Association.
Rural Nursing Association.

### Probationers.

Birmingham Children's Hospital.
Coventry City Hospital.
King's Lynn Hospital.
Liverpool Cancer Hospital.
Lowestoft Hospital, £12.
Nottingham Nursing Association.
Salford Union.
Workhouse Infirmary Association.

### Amusements and Relaxation.

### FOURTH QUARTERLY WORD COMPETITION

**Commenced Jan. 4th, 1890, ends March 29th, 1890.**

Three Prizes of 15s., 10s., 5s., will be given for the largest number of words derived from the words set for dissection.

Proper names, abbreviations, foreign words, words of less than four letters, and repetitions are barred; plurals, and past and present participles of verbs, are allowed. Nuttall's *Standard* dictionary only to be used.

N.B.—Word dissections must be sent in WEEKLY, arranged alphabetically, with correct total affixed.

The word for dissection for this, the FIFTH week of the quarter, being "PENSIONS."

### Results of Third Week.

| Names. | Jan. 23rd. | Totals. | Names. | Jan. 23rd. | Totals. |
|---|---|---|---|---|---|
| Holland | 38 | 147 | Nurse Clague | — | 40 |
| Red Cape | 32 | 94 | Lauriston | 39 | 175 |
| Nurse Marie | 29 | 122 | B. E. T. | 28 | 105 |
| Garfield | 36 | 161 | Jacko | 25 | 110 |
| Midget | 39 | 160 | Lady Clara | 40 | 161 |
| E. M. S. | 39 | 149 | Patience | 41 | 171 |
| Sister | 35 | 144 | Ecila | 40 | 160 |
| Duchess | — | 39 | Maude | 39 | 165 |
| Ellice | 40 | 162 | Esperance | 39 | 165 |
| Reldas | 41 | 168 | Ruby | 40 | 166 |
| Jenny Wren | 37 | 157 | M. W. | 40 | 165 |
| Mopus | — | 122 | Crystal Palace | 36 | 122 |
| Neptune | — | 118 | Jackanapes | 34 | 110 |
| Qu'appelle | 39 | 165 | M. M. M. | 25 | 25 |
| Whitehoe | 37 | 147 | | | |

### Notice to Correspondents.

N.B.—All letters referring to this page which do not arrive at 139 and 140, Salisbury-court, London, E.C., by the *first post on Thursdays*, and are not addressed PRIZE EDITOR, will in future be disqualified and disregarded.

